# An Observational Study on Play and Physical Activity Associated with a Recreational Facility-Led Park-Based “Loose Parts” Play Intervention during the COVID-19 Pandemic

**DOI:** 10.3390/children10061049

**Published:** 2023-06-12

**Authors:** Calli Naish, Gavin R. McCormack, Anita Blackstaffe, Levi Frehlich, Patricia K. Doyle-Baker

**Affiliations:** 1 Department of Community Health Sciences, Cumming School of Medicine, University of Calgary, Calgary, AB T2N 4N1, Canada; carol.naish1@ucalgary.ca (C.N.); levi.frehlich@ucalgary.ca (L.F.); 2 Department of Communication, Media and Film, Faculty of Arts, University of Calgary, Calgary, AB T2N 1N4, Canada; 3 Faculty of Kinesiology, University of Calgary, Calgary, AB T2N 1N4, Canada; pdoyleba@ucalgary.ca; 4 Faculty of Sport Sciences, Waseda University, Tokorozawa 359-1192, Japan; 5 School of Planning, Architecture, and Landscape, University of Calgary, Calgary, AB T2N 1N4, Canada; 6 Alberta Children’s Hospital Research Institute, University of Calgary, Calgary, AB T2N 4N1, Canada

**Keywords:** unstructured play, intervention, children, loose parts, physical activity

## Abstract

Play is a human right, yet opportunities for unstructured play are declining. The COVID-19 pandemic further reduced children’s play opportunities. We conducted an observational study of a novel community-based intervention (play hubs) that facilitated unstructured play by offering loose parts in parks (Calgary, Canada) during the pandemic. Our descriptive study included systematic observation using the System for Observing Children’s Activity and Relationships During Play (SOCARP) and Tool for Observing Play Outdoors (TOPO) to capture physical activity, play, and social and environment interactions among children participating in the play hubs for 10-weeks in 2021 (n = 160) and 2022 (n = 147). Play hub attendance was low. Most children observed were aged 5 to 12 years (2021: 93% and 2022 98%), with boys and girls represented (2021: 58% male/42% female and 2022: 52% male/48% female). Standing, sitting, and moderate activity were common activities. Physical, exploratory, and expressive play were common, while digital, bio, and rule-based play were less common. Children typically played alone or in small groups and engaged with loose parts or played in the open spaces. The play hubs encouraged unstructured play and promoted positive social interactions among children, despite the challenges of implementing a community-based intervention under pandemic public health restrictions.

## 1. Introduction

Play has long been established as a child’s right [1] reflecting its immediate and long-term social–emotional, cognitive, and physical health benefits [2,3,4]. Unstructured play (i.e., play that is self-determined and self-directed) contributes to the accumulation of physical activity, builds confidence, and develops children’s risk assessment skills, coping skills, and physical literacy [5,6]. Loose parts play (LPP) is a type of unstructured play that has gained popularity [6]. LPP involves offering children a variety of synthetic typical (e.g., sports equipment and toys) and atypical (e.g., spare parts, tools, utensils, and building supplies) and natural materials in which they interact with physically and cognitively in a self-determined and self-directed manner [6,7,8]. Playing with loose parts can help to maintain children’s attention, keeping them engaged in play for longer periods [9]. LLP can encourage physical activity [5] as well as creativity, discovery, and innovation [6,7,8]. Providing children with loose parts in environments that are safe, natural, and outdoors can foster diversity in children’s play activities [10,11,12,13]. Outdoor LLP may incorporate environmental features such as hills and trees to further facilitate unstructured play [14].

Studies investigating the effectiveness of LLP interventions on physical activity and play tend to be implemented within early education settings (e.g., childcare centres, preschools and elementary schools) [6,15,16,17,18]. However, studies investigating LLP interventions delivered to the public in community outdoor settings are lacking. Descriptive data about participation, physical activity, and play in loose parts interventions in public spaces (e.g., no constraints on who can participate) where there are fewer institutional or organizational regulations (e.g., school policies) are needed to inform future community delivered play interventions. Moreover, evidence from observational studies suggests that outdoor play declined during the COVID-19 outbreak during which access to recreational and play opportunities were severely restricted [19]. There is a dearth of studies on play interventions delivered during the first years of the COVID-19 pandemic.

We undertook a descriptive study of the Vivo Play Hub project—a community-based intervention that provided free, supervised unstructured play opportunities in local parks for children. The project was initiated by Vivo for Healthier Generations (a charitable enterprise in Calgary; Vivo). Vivo includes a large recreation centre in North Central Calgary (Alberta, Canada) which was involved the development and delivery of six temporary outdoor ‘play hubs’ where children were able to participate in LLP. Vivo implemented and in some cases modified the intervention between autumn 2019 and summer 2022, during the COVID-19 pandemic public health restrictions. The aim of our study was to undertake a descriptive analysis of children’s (1) physical activity and types of play and (2) social and environment interactions during play at the Vivo play hubs.

## 2. Materials and Methods

### 2.1. Play Hub Intervention

Vivo established six play hubs at parks in five north central Calgary communities (Beddington, Coventry, Hidden Valley, Huntington Hills, and Panorama) located within the recreational facility’s catchment area. The sociodemographic characteristics of the residents varied across the five communities (i.e., population 0–14 years of age: 17–25%; annual median household income: CAD 73,839–121,465; visible minorities: 30–69%; immigrants: 26–48%; and post-secondary education: 50–61%). Vivo’s choice of parks was based on feedback from local residents (Table 1). The parks ranged in size (0.6–14 ha), but most were smaller (median ~1.4 ha) and varied in their amenities (e.g., garbage cans and seating), presence of trees and lighting, level of graffiti, and connectedness to pathway and sidewalk networks (Table 1). All the parks included playground equipment and four parks included sport fields or courts. At all but one location (i.e., Hidden Valley), play materials were stored in large shipping containers, which were purposefully painted to increase the visibility of the play hubs and to help demonstrate the “messy” aspects of unstructured play. The shipping containers were incorporated as a fixed manufactured element within the play space. In addition to incorporating loose parts (Table 2), the play hubs often integrated natural (e.g., trees, hills) and fixed manufactured (e.g., playground equipment) environmental features.

At least two play ambassadors facilitated each play hub. Most play ambassadors had experience working with children. Play ambassadors received in-person and online training that covered content related to the importance of play, different types of play, strategies for facilitating play, and operation of the play hubs. The play hub ambassadors received remuneration for facilitating the play hubs. The play ambassadors were responsible for setting up the play hub space including the assortment of loose parts in a variety of configurations at each site, initiating a sign-in, and overseeing the safety and use of the play hubs. The play ambassadors configured the play hub in the same location within the park for each event.

**Figure 1:**
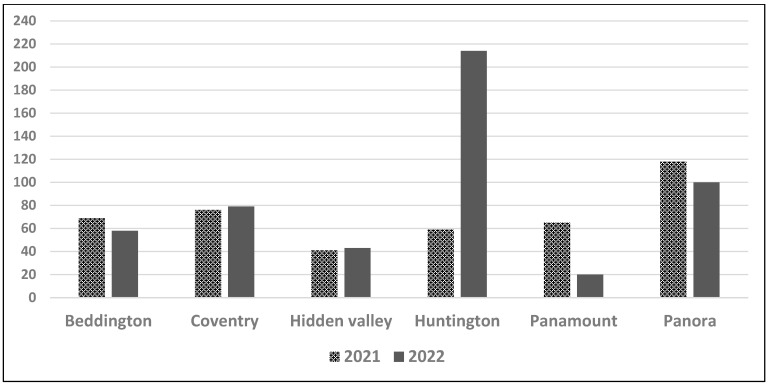
Registered child (5–12 years of age) attendance for all play hubs delivered in 2021 (July–September) and 2022 (April–June).

Vivo launched the play hubs in autumn, 2019, with an event offered in each park weekly. The intervention was initially scheduled to be delivered until summer, 2022; however, due to the pandemic, Vivo canceled some events (March–September 2020 and December 2021–February 2022). To comply with pandemic public health restrictions, Vivo modified the delivery of the play hub events until July 2021. During these modified events, physical distancing was enforced and facilitated through the segmenting of the play hub area (into smaller sub-areas) within which only members from the same family could play. After July 2021, play ambassadors encouraged participants to maintain physical distancing between families or households, and to mask if possible when playing within 2 metres of others. During the delivery of the play hub intervention (2019–2022), Vivo modified the times, days, and park locations of some play hubs for administrative reasons (e.g., staffing availability, community association requests, and scheduled park re-construction and renovation). Children could join the play hub event at any time. Based on the list of registered attendees collected by Vivo, the total number of children (5–17 years of age) attending play hubs between July and September 2021 was 420 (65 events; mean = 6.5 children/event) and between April and June 2022 was 511 (51 events, mean = 10.0 children/event) (Figure 1).

### 2.2. Study Design

Our study included a systematic observation of the play hub events that were delivered in summer, 2021 (July–September), and spring, 2022 (April–June). Play hubs held in Coventry, Beddington, and Hidden Valley were always offered on weekends, and play hubs held in Huntington Hills and Panora Square were always offered on weekdays. Vivo offered the play hub in Panamount Square on weekends in 2021 and weekdays in 2022. In 2022, Vivo relocated the play hub in Huntington Hills to a different park, adjacent to the community centre, to facilitate the ongoing delivery of the play events by the Huntington Community Association. Each play hub was 3.5–4 h in length and delivered between 11:00 and 18:00 on weekend days, and between 13:00 and 18:00 on weekdays (excluding Monday and Friday). Systematic observations were scheduled for each of the six play hub locations every second week for 10 weeks, with the goal of observing 30 events in 2021 and 2022. In situations where a play hub was cancelled (i.e., poor weather conditions or poor air quality) or where the play hub did not have any participants, systematic observations were re-scheduled to the following week. The University of Calgary Conjoint Health Research Ethics Board approved the study (REB20-0074).

### 2.3. Data Collection Procedure

The systematic observation captured children’s physical activity and play during the play hubs. We gathered data using the SOCARP (System for Observing Children’s Activity and Relationships During Play) [20] and the TOPO (Tool for Observing Play Outdoors) [11]. During the sign-in, the play ambassadors informed the caregivers of children attending the play hub that our team was collecting data.

Two trained research assistants (RAs) conducted systematic observations at each play hub. Prior to the start of the play hub, RAs photographed the loose parts materials and other park infrastructure (e.g., playgrounds and trees) integrated into the play area. The two RAs stood at a predetermined location that provided clear sightlines of the entire play space. The RAs recorded the weather conditions, time, and number of play ambassadors. Prior to selecting a child for observation, the RAs recorded the total number of children and parents within the play hub space. The RAs scanned the play space, alternating from right-to-left and left-to-right, to identify a child in their field of vision to observe. The RAs recorded their subjective assessment of the sex (male or female) and age (5–12 years or 13–17 years) of the selected child. The child’s activities were recorded following procedures described elsewhere [20]. The RAs observed the same child for 10 min, which included alternating between observation (10 s) and recording (10 s) (i.e., maximum of 30 recordings per child and up to six children observed per hour). During the observation, one RA recorded data using the SOCARP and the other RA recoded data using the TOPO, concurrently. The monitoring of a child that exited the play hub space was paused for one minute (no recording of activity undertaken outside the space), with observation resuming if the child returned. At the conclusion of the observation for a child, the RAs selected another child. The observation procedure was repeated for the duration of the play hub.

Four RAs in 2021 and six RAs in 2022 (four original and two new RAs) conducted the observations. RAs rotated through each of the play hub locations. All RAs participated in virtual and in-person training sessions that involved in-depth review of the two systematic observation tools. During these training sessions, the RAs reached consensus on the coding of unusual and obscure activities (e.g., crouching between sitting and standing and establishing rule-based play). RAs undertook in-person training during several play hubs that preceded the formal data collection period. The RAs participated in reliability testing sessions during which all RAs simultaneously administered the SOCARP and TOPO at two play hubs scheduled prior to the beginning of the formal data collection in 2021 and 2022.

### 2.4. Data Collection Tools

The SOCARP is a reliable tool for real-time recording of a child’s physical activity [20]. Initially developed for observing physical activity in school playgrounds, the tool is adaptable to other settings. The SOCARP captures a child’s activity level (laying, sitting, standing, moderate, and vigorous), activity type (sport, active game, sedentary, or locomotion), group size (along, small (2 to 4), medium (5 to 10), and large (>10)) and social interaction with others (none, verbal sportsmanship (e.g., offering support or praise), and physical sportsmanship (e.g., helping others, high five, holding hands, gently hugging another child, playing swords), physical conflict, and verbal conflict, and ignores negative interactions). Among the RAs (2021 and 2022 inclusive), the SOCARP had adequate inter-rater agreement (activity level = 91%; activity type = 86%; and group size = 95%).

The TOPO captures a child’s type of play and their interactions with the physical environment [11]. We used an abbreviated version of the TOPO (i.e., TOPO-9) to capture: (1) *physical play*, i.e., activities that test physical capabilities; (2) *exploratory play*, i.e., sensory-based explorations of an object or the environment; (3) *imaginative play*, which includes elements of imagination, role play, or play pretend; (4) *play with rules*, where at least two children have established a framework of rules of their activity; (5) *expressive play*, in which communication or expression is central to the play activity; (6) *bio play*, where the focus of the play is on an aspect of nature such as a plant or animal; (7) *restorative play*, which includes resting, retreating, or on-looking, and captures moments where children might be removed from but still engaged in the play; (8) *digital play*, i.e., play augmented by the use of digital technology; and (9) *non-play*, i.e., moments that occur in the outdoor play cycle where children are not engaged in play such as self-care (e.g., eating a snack), distress or conflict, or transitioning activities [11]. We also recoded a child’s interaction with the environment [21], including with fixed manufactured (e.g., playground), fixed natural (e.g., trees), loose manufactured (e.g., shovel and buckets), and loose natural (e.g., sticks and leaves) elements. We assigned ‘open’ when we observed no environmental interactions or where loose parts were obscured from view. We recorded up to two play types and five environmental interactions during the 10 s observation period. Among the RAs (2021 and 2022 inclusive) the TOPO had adequate inter-rater agreement (play type = 89% and environmental interaction = 96%).

### 2.5. Analysis

We estimated descriptive statistics (means and standard deviations and frequencies) for participation, child characteristics, and SOCARP and TOPO variables for play hub locations and year. The percentage of each SOCARP and TOPO variable was estimated by dividing the number of times each category was observed for a child (numerator) by the total number of complete observations for that child. We averaged these estimates across all play hub participants. Thus, the unit of analysis represents the percentage of observations for a given activity undertaken during the play hub. Independent *t*-tests were used to compare the mean proportions for SOCARP and TOPO variables between 2021 and 2022. Statistical analysis was undertaken using IBM SPSS Statistics for Windows (version 25; 2017). We used *p*-values of less than 0.05 as a criterion for reaching statistical significance.

## 3. Results

### 3.1. Play Hub Visitors

Systematic observation was completed for six play hubs at each of the park locations in 2021 and 2022 (n = 30 events total) (Table 3). On days that play hubs were monitored, the average minimum and maximum daytime temperatures were 10.9 °C and 24.1 °C in 2021 and 2.3 °C and 14.5 °C in 2022, respectively. The average precipitation was 1.97 cm (six rain days only) in 2021 and 1.17 cm (three rain days and two snow days) in 2022. In 2022, we observed snow accumulation on the ground at three play hub events.

In 2021, the average total number of children and adults attending the play hubs observed at any one time was five and two, respectively (Table 3). The average attendance was similar in 2022, where the average total number of children and parents observed in the play hubs at any one time was seven and three, respectively. The number of children attending the play hubs at any one time ranged from 1 to 13 in 2021, and from 1 to 48 in 2022. The number of adults in attendance ranged from 0 to 8 in 2021, and 0 to 15 in 2022 (Table 3).

We collected observation data on 160 and 147 children (5–17 years of age) in 2021 and 2022, respectively. The number of children observed at each play hub was consistently low in both 2021 and 2022 (Table 3). The majority of children attending the play hubs were 5 to 12 years of age (2021: 93% and 2022: 98%). The sex distribution of children attending the play hubs was similar in 2021 (male: 58%/female: 42%) and 2022 (male: 52%/female: 48%), although this varied by play hub location. For play hubs in Beddington (2021 and 2022) and Panamount Square (2022), the majority of the children were female (Table 3). Between 83% and 100% of the children observed were monitored for the entire 10 min period (Table 3). Children identified with partially complete observations included those who had exited the designated play hub space while being monitored. Given the low number of children attending each play hub at any given time, we were able to observe most children who attended.

### 3.2. SOCARP Variables

Across most park locations (except for Panamount Square, 2021, Coventry, 2022, and Panora Square, 2022), standing (2021: 43.5%/2022: 44.5%), moderate activity (2021: 28.2%/2022: 30.0%), and sitting (2021: 18.7%/2022: 14.9%) were the most observed behaviors undertaken by children (Table 4). Lying down (2021: 0.4%/2022: 0.9%), followed by vigorous activity (2021: 9.3%/2022: 9.6%), were the least observed behaviors overall. In 2021, the most common activity observed during the play hubs was sedentary (49.9%), followed by locomotion (25.3%), games (23.7%), and sports (1.1%). However, in 2022, the most common activities observed during the play hubs was games (55.8%), followed by sedentary (24.0%), locomotion (13.7%), and sports (6.5%) (Table 4).

Across all park locations and years, most children participated in play in small groups (2021: 74.9%/2022: 69.0%) or alone (2021: 19.8%/2022 24.1%). Play in medium and large groups was less common. Over three-quarters of observations involved no interactions between children (2021: 77.3%/2022: 77.9%); however, among the interactions observed, most included verbal (2021: 16.7%/2022: 15.3%) and physical (2021: 4.7%/2022: 6.2%) sportsmanship. Few physical or verbal conflicts were observed (<0.05% in 2021 and 2022) (Table 4).

### 3.3. TOPO Variables

The three most common play types observed included physical (2021: 28.5%/2022: 29.3%), exploratory (2021: 26.6%/2022: 28.7%), and expressive (2021: 14.5%/2022: 13.7%) play (Table 5). The three least common play types were digital (2021: 0.04%/2022: 0%), bio (2021: 0.5%/2022: 0.2%), and rule-based (2021: 2.8%/2022: 5.5%). In 2021 and 2022, approximately 7% of observations were non-play (e.g., self-care activities).

The majority of play observed involved children interacting with loose manufactured materials (2021: 66.7%/2022: 70.1%) or no materials (2021: 16.8%/2022: 17.0%). Despite contributing to a smaller proportion of observations, interactions with fixed manufactured, loose natural, and fixed natural materials were also observed, but varied, reflecting the availability of infrastructure (e.g., playgrounds and trees) across parks and the integration of this infrastructure into the play hub space (Table 5).

## 4. Discussion

Our study captured physical activity and types of play, and social and environment interactions among children participating in a community-based intervention (play hubs) that encouraged unstructured play via loose parts in local parks. Our findings suggest that children attending the play hubs interacted with the loose parts, resulting in participation in different play types, especially physical, exploratory, and expressive play, and in different levels of physical activity. The play hubs encouraged social interaction and facilitated play that embodied creativity and discovery [7,8].

Congruent with previous findings [5], loose parts available during the play hubs encouraged physical activity. While the play hubs promoted different types of play, they also encouraged different intensities and types of physical activity. Approximately 40% of the observed activity undertaken was of moderate to vigorous intensity. The play hubs facilitated the accumulation of physical activity and could potentially support children in achieving the 60 min of MVPA daily needed to promote health [22]. The Canadian 24 h movement guidelines suggest that children can accumulate physical activity by engaging in unstructured and structured light physical activities [22]. The play hubs’ facilitation of outdoor unstructured physical activity is encouraging given that children who increased their time spent outdoors during the pandemic were more likely to meet MVPA guidelines compared with children who decreased their outdoor activity [23]. However, we found that the play hubs also encouraged sedentary activities. For example, children’s imaginative play (e.g., playing kitchen or house) primarily involved sitting and standing. The loose parts provided opportunities for unstructured play that could be active or sedentary, depending on how children decided to use the materials and space. The type of loose parts available can shape children’s play decisions and experiences [16]. The play hubs aimed to encourage child-led unstructured play rather than specifically promoting physical activity.

Different contexts can encourage specific types of behavior. The play hubs represent a behavior setting [24,25] in which the physical space (e.g., loose parts, location within park, presence of park infrastructure, weather), social space (e.g., children, parents, and play ambassadors), and temporality (e.g., during pandemic, time and days of scheduled events) shaped the children’s play and physical activity. We observed that children playing with the same loose parts did so in different ways, reflecting their personal preferences, imagination, creativity, and physical and cognitive abilities. The loose parts provided a range of options for play, supporting inclusivity in terms of offering opportunities for different types of play that appealed to a broad range of children with different preferences, backgrounds, and experiences [26]. Our finding that most environmental interactions observed during the play hubs involved loose manufactured features and open areas reflects the specificity of the intervention in terms of encouraging unstructured play (i.e., the observed behaviors were logically and empirically linked with the exposure). For instance, traditional play equipment (e.g., balls and bats) contributed only a small proportion of the loose parts provided, resulting in less sport and vigorous activities during the play hubs. The choice of the types of loose parts offered to children should be informed by the specific goals of the intervention (i.e., to promote physical activity specifically or to promote play generally).

The play hubs provided an opportunity for children to play outdoors in a safe, supervised environment. Exposure to outdoor environments, including green and natural spaces, offer children physical, emotional, mental, and cognitive benefits [27]. The play hubs provided children with a space to socialize, which is important given that opportunities for socializing with friends and peers among children has been negatively impacted during the pandemic [28,29]. We found that over two-thirds of the activity observed in the play hubs involved play among small groups of children. Further, the nature of most of the observed social interactions between the children at the play hubs was positive. Evidence elsewhere suggesting that LLP may encourage co-operative behaviors corroborates our observation of mostly positive social interactions among children during the play hubs [30].

The number of children attending the play hub events was lower than might be expected given that the community-based intervention was free to attend and scheduled on days and times that children would be available to attend (e.g., after school on weekdays and on weekends). Vivo promoted the play hubs via social media (i.e., Facebook and Instagram), outdoor banners, and the brightly painted storage containers (sea-cans). Despite Vivo’s efforts to promote the play hubs, the low attendance could have been due to a lack of community awareness. In a 2020 survey of north central Calgary community households, approximately one-quarter of adults reported they were aware of any of Vivo’s community-based programs (prompted recall) and about 8% reported being aware of the play hubs [31]. Elsewhere, parents reported being less aware of recreational programs that were available during the pandemic [32]. The attendance could reflect public anxiety related to the pandemic [33,34]. The average play hub attendance of children 5–17 years of age increased from 2021 to 2022, possibly reflecting reductions in parent anxiety or fear related to COVID-19 transmission. Despite the easing of public health pandemic restrictions, many parents likely remained apprehensive about allowing their children to participate in recreational programs due to safety concerns regarding disease transmission [32].

Our study has several strengths, including the use of an established systematic observation approach and tools to collect data over multiple years. Our study is novel as it included data collected for a community-based intervention implemented during the COVID-19 pandemic. However, we acknowledge several limitations of our study. We took steps to minimize participant reactivity by locating the RAs outside the boundary of the play hub space while still maintaining clear sightlines; however, we cannot rule out children modifying their behavior in response to observation. Further, the need to maintain distance between the observers and play hub participants may have negatively affected the accuracy of the behaviors or activities recorded. The RAs found SOCARP relatively straightforward to administer; however, they initially found administering TOPO challenging. Deciding between different types of play, reporting multiple types of play, and recording play interactions with multiple environmental features in real time required significant attention on behalf of the RAs. When feasible, video recording play events and then coding play using the TOPO may improve the accuracy of the results. Moreover, the recording of a child’s sex and age based entirely on observation is prone to error. Our findings regarding sex and age should therefore be interpreted with caution. Researchers should use other approaches for gathering participant’s personal information (gender, sex, age, ethnicity, etc.) to complement the data gathered via systematic observation. Given that children self-selected to attend the play hubs, we also cannot rule out that these included highly motivated children (or parents) that would have sought out other play opportunities if the play hubs were not available. Our study does not allow us to infer reasons as to why children attended the play hubs nor their personal experiences of participating in the intervention. We did not record data on play ambassador supervisory or facilitation style or on the interactions between the play ambassadors and children during the play hubs. While we cannot rule out the potential influence of play ambassadors in shaping children’s activities during the play hubs, all play ambassadors received play training and had previous experience working with children. Given the intervention’s aim of facilitating child-led unstructured and risky play, play ambassadors were instructed to minimize their interactions with children and to only intervene when necessary (e.g., to minimize hazards or resolve unsafe situations).

## 5. Conclusions

The community-based play hubs, designed and implemented by a local recreational facility to increase unstructured LLP, facilitated different types of play and physical activity and fostered positive social interactions among children. The play hubs offered children an opportunity to participate in unstructured play despite the challenges of navigating the changing public health pandemic restrictions. Future research incorporating experimental study designs are needed to estimate the effects of play hubs and similar unstructured outdoor LLP interventions on changes in children’s play and physical activity. Programs offering supervised unstructured play in local outdoor public spaces may be one approach to combating the decline in play among children.

## Figures and Tables

**Table 1:** Community Park Descriptions for Play Hub Locations.

Park	Description	Images Illustrating the Play Hub Areas and Loose Parts
Beddington 2021 Sundays 10:30–14:30 2022 Sundays 11:30–15:00	The Beddington Play Hub was located at a small (~0.6 ha) neighbourhood park within a cul-de-sac that was geographically disconnected from community pathways and sidewalks and major roads. The park included a maintained, level, grassed area, which included a playground, benches, picnic table, and trash can. A small hill connected to the leveled grassed area was located at the park boundary adjacent to the cul-de-sac. The park consisted of a few trees that provided limited shade, located along the parks border and adjacent to the playground. The playground featured structures to slide down, climb on/up/through, swing on, and dig in. The playground area also included a teeter-totter, spring toys, and tire swing. There was no lighting within or outside the perimeter of the park. Signage was not present in the park. No graffiti was observed on any park structures. Play hub activities were permitted in all areas of the park but were primarily contained to the leveled grassed area and playground. Materials were connected to trees in the play space to encourage swinging, climbing, and ziplining.	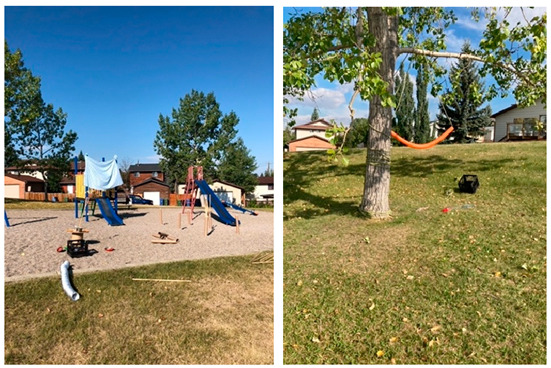
Coventry 2021 Saturdays 10:30–14:30 2022 Saturdays 11:30–15:00	The Coventry Play Hub was located at a large park (~14 ha) adjacent to a main road. The park was located on the outskirts of the community and extended beyond the community boundary. The park consisted of two main areas, the largest of which included an un-maintained, ‘natural’ landscape and the smallest of which included maintained, level, grassed area with one small hill on the periphery. Trees offering shade were situated along the park’s perimeter and adjacent to the regional pathway running through its centre. The smaller grassed area included a playground providing children with structures to slide down, climb up/on/through, swing on, and dig in. This smaller area also included benches and trashcans as well as street lighting along its perimeter. An aesthetic stone archway and sign for “Nose Creek Park” marked the entrance to the park and regional pathway. Graffiti was observed on some park structures. Play hub activities were contained to the area surrounding and including the playground equipment for some events, as well as a small hill adjacent to the playground.	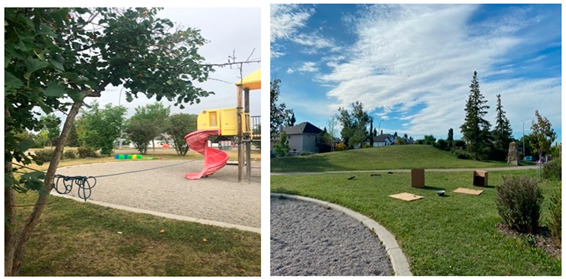
Hidden Valley 2021 Sundays 14:00–18:00 2022 Saturdays 11:30–15:00	The Hidden Valley Play Hub was located at a mid-sized park adjacent to two schools, an elementary school, and a middle school. The park (~8.7 ha) was adjacent to a main road. Pathways crossed through the park connecting with community pathways and sidewalks. Streetlights were located on the park’s periphery adjacent to the surrounding sidewalk. Apart from a few small trees planted adjacent to the tennis court and surrounding the soccer field, the majority of the park was without trees or shade. Bicycle storage was available next to the parking lot of the middle school. The park consisted of level sporting areas (tennis courts, baseball diamonds, and a soccer field) separated by small hills. A basketball court was adjacent to the middle school. A large hill was located opposite the schools at the farthest end of the park. A playground was located next to the elementary school offering children structures to slide down, climb on/up/through, swing on, and dig in, as well as a rock-climbing wall. Graffiti was observed on some park structures. Signage pertaining to dog-leashing, tennis court management, and hazardous flooding were present in the park. The play hub activities were contained to the centre of the park (between the sports fields) and did not include the playground or sporting areas.	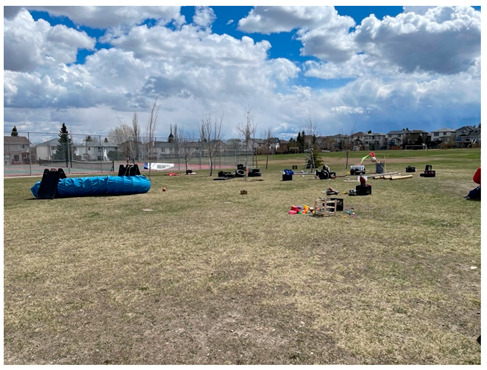
Huntington (2021) Thursdays 14:00–18:00	In 2021, the Huntington Play Hub was located at a park situated within a cul-de-sac. This small, maintained park (~0.8 ha) consisted of an open level grassed area with minimal shade offered by the bordering trees. The park was disconnected from the surrounding community pathways and sidewalks. The park included a playground that provided children with structures to slide down, climb up/on/through, swing on, and dig in. The playground was adjacent to street lighting located at the park’s perimeter. The park included benches and a trashcan. Signage was not present in the park. Graffiti was not observed on any park structures. The play hub activities utilized the entire park, including the playground. Materials were connected to trees in the play space to encourage swinging, climbing, and ziplining.	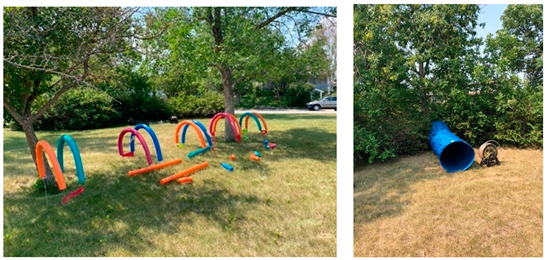
Huntington (2022) Wednesdays 14:00–17:30	In 2022, the Huntington Play Hub was relocated to a larger park (~7 ha) situated at the corner of two intersecting major roads and adjacent to the Huntington Hills Community Association’s building, playground, and parking lot. A roadway providing access the community association crossed the park. A regional pathway crossed through the unfenced off-leash area at the north side of the park, connecting back to the sidewalk that bordered the perimeter of the park. Lighting was situated along the regional pathways and sidewalks surrounding the park. The park included a large hill rising from the community association parking lot to the north side of the park. This hill was divided into smaller hill sections by leveled grassed areas that included two baseball diamonds. Trees along the pathways and surrounding the baseball diamonds provided limited shade. Graffiti was observed on some park structures. Signage pertaining the baseball diamonds (i.e., rules of use) and dog off-leash areas were present in the park. Public washrooms were available inside the community association during operational hours. The play hub activities were contained to the small hill and leveled grassed area adjacent to the community association parking lot and included the playground as part of the play space. Sporting areas were not included in the play space.	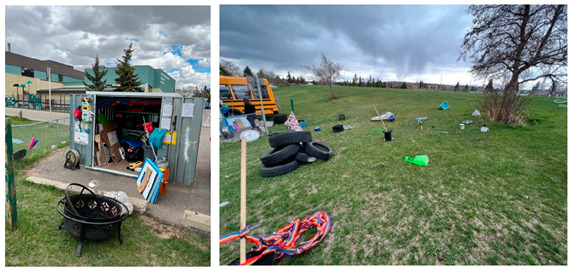
Panamount Square 2021 Saturdays 15:00–19:00 2022 Tuesdays 14:00–17:30	The Panamount Square Play Hub was located at a small (~1.4 ha), maintained park adjacent to a main road. The park consisted of a two level, open, grassed areas divided by a playground, basketball court, and a pathway, which connected to surrounding community pathways and sidewalks. A large archway was present at the entrance to the park. Street lighting was adjacent to the sidewalk bordering the park’s perimeter. Trees bordering the pathway and park perimeter provided minimal shade. A large circle of trees encircled a playground area. Two playground apparatus, adjacent to each other, provided children with structures to slide down, climb up/on/through, swing on and dig in. A shaded gazebo with picnic tables offered a sightline to the playground area. A fire pit and signage (i.e., rules of use) was available at the edge of the park. Graffiti was observed on some park structures. The play hub was contained to the open grassed areas and the playground, excluding the basketball court. Materials were connected to trees in the play space to encourage hiding and building (e.g., shelters).	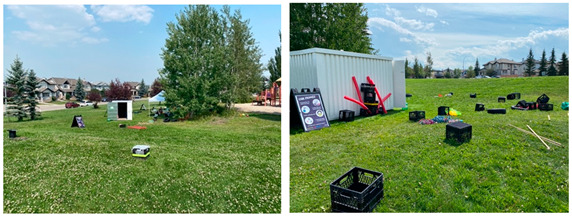
Panora Square 2021 Wednesdays 14:00–18:00 2022 Thursdays 14:00–17:30	The Panora Square Play Hub was located at a small (~0.8 ha) maintained and landscaped park adjacent to a main road. A large stone archway and two large planters containing flowers marked the park entrance. A pathway crossing through the park divided the level, open grassed area. A playground, basketball court, gazebo with a picnic table, were located to one-side of the pathway, while an open green space was located to the other-side. Trashcans and benches were located along the pathway. This pathway connected to community paths and the sidewalks. Streetlights were located alongside the surrounding paths and sidewalks. Trees bordering the park perimeter, grassed area, and pathway provided limited shade. The playground provided children with structures to slide down, climb up/on/through, swing on, and dig in, as well as a rock-climbing wall. Signage was not present in the park. Graffiti was observed on some park structures. The play hub activities were contained to the open grassed area to one-side of the park and did not include the playground or basketball court. Materials were connected to trees in the play space to encourage hiding and building (e.g., shelters).	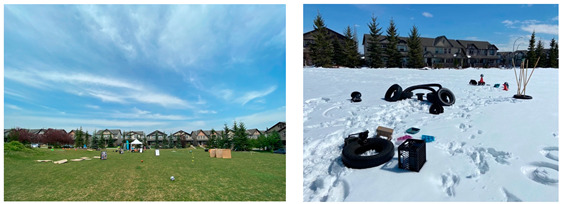

**Table 2:** Examples of loose parts offered during the play hub events.

Loose Parts	Examples
Kitchen utensils	Bowls, tongs, spatula, funnels, pots, measuring cups, spoons, pans, silicone muffin tins
Garden equipment	Hand-hoe, hand rake, hand shovel, snow shovel, rake
Hand tools	Hammers, screw drivers, mallets
Sports equipment	Soccer ball, basketball, volleyball, soccer nets, frisbee, football
Toys and games	Hula hoops, pool noodles, magic carpets, horseshoe, skipping rope, tunnels, costumes/dress-up
Other equipment	Wheels, pulleys, mallet, buckets, climbing rope, PVC pipe, car/bike tires, milk crates, inoperable electronics (old laptops, camcorders), tarps
Consumables	Chalk/spray chalk, flagging tape, masking tape, cardboard/cardboard boxes, duct tape
Additional elements	Fire/fire pit, water, ice/ice blocks

**Table 3:** Play hub event, observations, and participant characteristics in 2021 and 2022.

	2021							2022						
	BED	COV	HID	HUN	PAN	PANSQ	Total	BED	COV	HID	HUN	PAN	PANSQ	Total
**Play Hub events observed**														
Total events	7	5	5	5	6	5	32	5	5	7	5	8	6	34
Events (no participants)	2	0	0	0	1	0	2	0	0	2	0	3	1	4
Events (with participants)	5	5	5	5	5	5	30	5	5	5	5	5	5	30
Event attendees *														
Children: average/event	5	4	3	4	5	6	5	4	5	5	7	3	13	7
Children: range/event	1–13	1–7	1–7	1–7	1–8	3–7	1–13	1–10	1–10	1–8	1–22	1–5	1–48	1–48
Adults: average/event	2	3	2	2	2	2	2	3	2	3	2	2	5	3
Adults: range/event	0–8	1–8	1–3	0–5	1–4	0–5	0–8	0–6	1–5	0–5	0–6	1–3	0–15	0–15
**Observed participants**														
Total	31	35	13	26	21	34	160	20	22	22	30	12	41	147
Range/event	1–9	4–12	1–5	2–8	2–7	4–9	1–12	1–7	2–8	3–5	3–9	1–4	2–12	1–12
Average/event	6	7	3	5	4	7	5	4	4	4	6	2	8	5
Complete observations (%) **	90	89	100	89	81	85	88	100	91	96	83	92	85	90
**Sex**														
Male (%)	32	63	85	65	52	62	58	35	46	68	50	33	63	52
Female (%)	68	37	15	35	48	38	43	65	55	32	50	67	37	48
**Age group**														
5–12 years (%)	100	91	100	96	81	88	93	100	100	86	100	100	100	98
13–17 years (%)	0	9	0	4	19	12	7	0	0	14	0	0	0	2

* Estimates based on counts of children or adults recorded at any one time during a single play hub event. ** Complete observations defined as a child who is monitored for the entire 10 min (maximum of 30 × 10 s reporting intervals). BED: Beddington; COV: Conventry; HID: Hidden Valley; HUN: Huntington; PAN: Panamount Square; PANSQ: Panora Square.

**Table 4:** Percentage of observed SOCARP activities (all play hubs combined) in 2021 and 2022.

	2021							2022						
	BED M (SD)	COV M (SD)	HID M (SD)	HUN M (SD)	PAN M (SD)	PANSQ M (SD)	*Total* *M* (*SD)*	BED M (SD)	COV M (SD)	HID M (SD)	HUN M (SD)	PAN M (SD)	PANSQ M (SD)	*Total* *M* (*SD*)
**Activity Level**														
Standing	**36.0 (21.5)**	**42.6 (24.1)**	**52.6 (18.5)**	**44.3 (22.6)**	**49.3 (18.0)**	**43.6 (23.9)**	**43.5** **(22.6)**	**40.5 (21.3)**	**45.1 (18.2)**	**48.0 (19.7)**	33.0 (20.1)	**46.6 (23.2)**	**52.2 (19.9)**	**44.5 (21.0)**
Moderate	26.1 (18.8)	26.9 (18.9)	24.9 (17.4)	31.1 (20.3)	27.7 (13.0)	30.8 (18.1)	28.2 (18.0)	31.8 (17.7)	27.3 (10.7)	27.0 (17.0)	**36.4 (23.6)**	23.3 (15.1)	29.6 (17.7)	30.0 (18.1)
Sitting	28.4 (26.8)	24.7 (29.3)	12.8 (20.0)	17.6 (17.2)	7.5 (10.8)	13.5 (27.0)	18.7 (24.7)	22.0 (26.1)	11.5 (13.4)	14.2 (11.9)	19.8 (23.8)	20.3 (29.8)	8.4 (15.2)	14.9 (20.1)
Vigorous	8.4 (12.1)	5.5 (11.4)	9.2 (10.8)	6.9 (11.0)	15.0 (15.0)	12.1 (13.5)	9.3 (12.6)	5.7 (7.4)	15.8 (22.2)	10.8 (12.3)	6.8 (9.1)	9.4 (14.2)	9.7 (11.1)	9.6 (13.2)
Lying Down	1.1 (4.0)	0.3 (1.7)	0.5 (1.3)	0.1 (0.7)	0.3 (1.0)	0 (0)	0.4 (2.0)	0 (0)	0.3 (1.0)	0 (0)	4.1 (13.2)	0.3 (1.0)	0.1 (0.5)	0.9 (6.1)
**Group Size**														
Small	**74.0 (27.6)**	**70.9 (25.2)**	**67.7 (42.6)**	**87.2 (25.9)**	**76.9 (19.7)**	**71.8 (27.4)**	**74.9** **(27.6)**	**78.8 (25.1)**	**75.1 (21.9)**	**65.7 (30.2)**	**63.0 (28.2)**	**67.3 (33.4)**	**67.5 (26.8)**	**69.0 (27.4)**
Alone	18.3 (25.9)	26.8 (24.1)	7.2 (12.7)	12.8 (25.9)	19.6 (16.6)	24.6 (26.7)	19.8 (24.2)	19.5 (24.7)	18.8 (16.0)	32.3 (30.6)	27.8 (27.1)	27.5 (33.1)	21.1 (24.2)	24.1 (25.8)
Medium	6.9 (18.0)	2.4 (6.7)	25.1 (42.4)	0 (0)	3.5 (13.3)	3.6 (13.3)	5.1 (17.5)	1.7 (6.1)	6.1 (17.0)	2.0 (5.7)	9.1 (22.2)	5.3 (18.3)	10.9 (18.7)	6.8 (16.8)
Large	0.9 (3.4)	0 (0)	0 (0)	0 (0)	0 (0)	0 (0)	0.2 (1.5)	0 (0)	0 (0)	0 (0)	0 (0)	0 (0)	0.5 (3.1)	0.1 (1.6)
**Activity Type**														
Games	21.0 (25.5)	20.4 (24.1)	30.3 (27.1)	15.6 (17.8)	**40.3 (28.2)**	22.9 (22.4)	23.7 (24.7) *	**46.0 (26.7)**	**68.7 (28.2)**	**56.6 (39.6)**	**52.0 (29.7)**	**56.7 (31.0)**	**55.7 (35.7)**	55.8 (32.7) *
Sedentary	**53.1 (26.7)**	**58.8 (27.9)**	**55.4 (27.3)**	**51.8 (26.9)**	36.8 (23.0)	**42.4 (29.8)**	**49.9** (27.9) *	36.2 (24.0)	15.4 (13.2)	18.5 (26.3)	31.0 (24.2)	30.0 (29.2)	18.9 (25.4)	24.0 (24.8) *
Locomotion	24.1 (16.3)	20.2 (19.3)	32.1 (17.6)	32.1 (17.6)	21.0 (15.8)	33.6 (25.5)	25.3 (19.9) *	17.8 (9.4)	13.5 (20.7)	9.7 (6.9)	11.2 (7.6)	13.1 (9.8)	15.8 (16.6)	13.7 (13.5) *
Sports	1.7 (4.9)	0.5 (3.2)	1.0 (2.8)	0.4 (1.4)	1.9 (8.7)	1.1 (4.7)	1.1 (4.7) *	0 (0)	2.4 (8.4)	15.2 (31.3)	5.8 (20.1)	0.3 (1.0)	9.5 (23.6)	6.5 (20.2) *
**Interactions**														
None	**74.6 (28.7)**	**84.8 (20.0)**	**89.9 (14.0)**	**83.8 (18.3)**	**59.6 (25.9)**	**73.3 (26.2)**	**77.3** **(24.8)**	**82.0 (25.7)**	**59.1 (33.9)**	**82.9 (25.4)**	**84.0 (21.2)**	**82.5 (14.7)**	**77.3 (30.2)**	**77.9 (27.7)**
Verbal sportsmanship	17.6 (27.1)	10.0 (16.5)	9.1 (13.2)	11.5 (17.5)	31.3 (24.4)	20.6 (24.2)	16.7 (22.5)	15.6 (23.2)	28.7 (25.0)	9.9 (16.8)	9.9 (15.6)	13.5 (13.9)	15.5 (22.1)	15.3 (20.9)
Physical sportsmanship	7.7 (12.6)	3.3 (5.8)	0 (0)	4.6 (8.9)	3.4 (5.8)	6.1 (10.4)	4.7 (9.0)	1.5 (3.4)	10.9 (13.0)	7.1 (10.1)	4.8 (8.1)	4.0 (6.1)	7.0 (12.5)	6.2 (10.3)
Physical conflict	0.1 (0.6)	1.3 (3.8)	0 (0)	0.1 (0.7)	3.4 (7.8)	0 (0)	0.8 (3.5)	0.5 (1.5)	0.4 (1.5)	0 (0)	1.0 (2.8)	0 (0)	0.1 (0.5)	0.3 (1.5)
Verbal conflict	0 (0)	0.3 (0.9)	0.5 (1.8)	0 (0)	1.5 (3.7)	0 (0)	0.3 (1.5)	0.5 (1.5)	0.7 (1.3)	0.1 (0.6)	0.3 (1.0)	0 (0)	0.2 (1.0)	0.3 (1.1)
Ignores	0 (0)	0.2 (1.4)	0.5 (1.8)	0 (0)	0.8 (2.0)	0 (0)	0.2 (1.1) *	0 (0)	0.1 (0.7)	0 (0)	0 (0)	0 (0)	0 (0)	0.0 (0.3) *

M(SD): Mean and Standard Deviation (percentage of observations for each category per child averaged over the number Play Hub events). **Bolded values:** The most common behavior observed. BED: Beddington; COV: Conventry; HID: Hidden Valley; HUN: Huntington; PAN: Panamount Square; PANSQ: Panora Square. * Statistically significant *p* < 0.05 (Independent *t*-test comparing 2021 and 2022 totals).

**Table 5:** Percentage of observed TOPO activities and environmental interactions (all play hubs combined) in 2021 and 2022.

	2021							2022						
	BED M (SD)	COV M (SD)	HID M (SD)	HUN M (SD)	PAN M (SD)	PANSQ M (SD)	*Total* *M* (*SD*)	BED M (SD)	COV M (SD)	HID M (SD)	HUN M (SD)	PAN M (SD)	PANSQ M (SD)	*Total* *M* (*SD*)
**Play types**														
Physical	**27.0 (13.0)**	28.7 (14.0)	**30.9 (11.2)**	27.2 (14.3)	**32.6 (11.8)**	**27.3 (12.6)**	**28.5 (13.0)**	22.9 (12.0)	**30.2 (9.4)**	29.3 (15.0)	**34.2 (12.0)**	26.4 (13.5)	**29.0 (10.4)**	**29.3 (12.1)**
Exploratory	25.4 (15.8)	**28.9 (14.0)**	24.6 (14.1)	**30.5 (15.0)**	21.9 (15.1)	26.4 (13.2)	26.6 (14.5)	**32.5 (9.9)**	28.4 (12.6)	**34.2 (14.8)**	27.9 (15.3)	**29.6 (14.6)**	24.4 (15.3)	28.7 (14.3)
Expressive	16.9 (11.9)	16.1 (13.3)	11.9 (12.3)	15.5 (20.6)	10.2 (10.1)	13.5 (10.4)	14.5 (13.5)	15.0 (13.3)	16.1 (10.8)	15.6 (14.1)	10.2 (9.2)	15.7 (11.7)	12.8 (10.0)	13.7 (11.3)
Restorative	12.3 (11.2)	11.5 (11.0)	11.6 (12.3)	11.6 (10.8)	10.2 (7.0)	9.3 (7.3)	11.0 (9.9)	10.2 (9.6)	5.7 (0.0)	6.1 (8.4)	10.3 (14.0)	11.4 (10.1)	11.7 (9.9)	9.4 (10.3)
Imaginative	6.9 (12.7)	4.9 (9.8)	0.6 (1.5)	8.0 (10.7)	12.5 (17.1)	13.6 (16.1)	8.3 (13.3)	9.9 (14.2)	6.5 (8.9)	3.8 (6.9)	3.8 (9.7)	10.1 (12.1)	4.7 (8.5)	5.8 (10.1)
Non-play	5.9 (7.1)	8.8 (10.4)	10.3 (9.4)	6.1 (8.4)	9.7 (10.8)	7.1 (6.1)	7.7 (8.7)	7.6 (9.4)	6.9 (11.9)	6.4 (7.9)	5.6 (7.9)	5.3 (7.3)	9.6 (11.1)	7.3 (9.7)
With Rules	4.5 (10.5)	0.9 (3.3)	9.7 (10.8)	0.2 (0.8)	2.9 (9.5)	2.6 (5.5)	2.8 (7.5) *	1.9 (4.2)	5.6 (8.9)	4.1 (7.3)	7.8 (14.4)	1.0 (2.5)	7.7 (13.8)	5.5 (11.0) *
Bio	1.2 (5.9)	0.2 (0.9)	0.0 (0.0)	0.9 (3.3)	0 (0)	0.1 (0.9)	0.5 (3.0)	0.0 (0.0)	0.4 (1.9)	0.4 (1.8)	0.1 (0.3)	0.4 (1.5)	0.1 (0.6)	0.2 (1.1)
Digital	0 (0)	0 (0)	0.3 (1.0)	0 (0)	0 (0)	0.1 (0.4)	0.0 (0.3)	0 (0)	0 (0)	0 (0)	0 (0)	0 (0)	0 (0)	0 (0)
**Environmental ** **Interactions**														
Loose manufactured	**57.5 (26.6)**	**50.6 (33.2)**	**78.5 (17.1)**	**69.2 (26.7)**	**66.9 (23.1)**	**85.1 (14.5)**	**66.7 (27.8)**	**66.9 (24.3)**	**52.5 (26.4)**	**85.5 (12.5)**	**63.1 (23.9)**	**75.7 (18.7)**	**76.2 (24.5)**	**70.1 (24.7)**
Open space	16.0 (14.2)	13.0 (16.8)	17.0 (14.2)	20.3 (23.5)	21.0 (16.3)	12.9 (13.5)	16.1 (16.8)	17.3 (15.5)	17.6 (21.6)	13.0 (11.1)	18.8 (24.3)	11.5 (12.9)	19.0 (23.4)	17.0 (20.1)
Fixed manufactured	11.2 (20.4)	28.7 (33.7)	0.0 (0.0)	1.7 (8.5)	4.1 (12.2)	0.0 (0.0)	9.3 (21.8)	5.2 (10.3)	23.6 (28.1)	0.1 (0.7)	5.8 (11.9)	4.0 (13.8)	0.0 (0.0)	5.8 (15.3)
Loose Natural	12.1 (16.3)	6.6 (13.1)	4.0 (12.9)	5.4 (10.4)	6.9 (12.8)	0.4 (1.8)	6.0 (12.3)	7.4 (14.2)	1.8 (5.4)	0.7 (3.1)	6.4 (12.5)	5.7 (12.2)	4.0 (10.2)	4.3 (10.4)
Fixed Natural	3.2 (9.4)	1.1 (3.2)	0.5 (1.2)	3.4 (8.4)	1.0 (4.1)	1.6 (5.2)	1.9 (6.3)	3.2 (7.0)	4.5 (10.9)	0.7 (3.1)	6.0 (12.5)	3.1 (5.0)	0.7 (2.3)	2.8 (8.0)

M(SD): Mean and Standard Deviation [percentage of observations for each category per child averaged over the number Play Hub events]. **Bolded values:** The most common behavior observed. BED: Beddington; COV: Conventry; HID: Hidden Valley; HUN: Huntington; PAN: Panamount Square; PANSQ: Panora Square. * Statistically significant *p* < 0.05 (Independent *t*-test comparing 2021 and 2022 totals).

## Data Availability

The data presented in this study are available on request from the corresponding author.
